# *Schizophyllum commune* induces IL-17-mediated neutrophilic airway inflammation in OVA-induced asthma model mice

**DOI:** 10.1038/s41598-019-55836-x

**Published:** 2019-12-18

**Authors:** Jun Hanashiro, Yasunori Muraosa, Takahito Toyotome, Koichi Hirose, Akira Watanabe, Katsuhiko Kamei

**Affiliations:** 1 Division of Clinical Research, Medical Mycology Research Center, Chiba University, Chiba, Chiba, Japan; 2 Department of Veterinary Medicine, Obihiro University of Agriculture and Veterinary Medicine, Obihiro, Hokkaido Japan; 3 Diagnostic Center for Animal Health and Food Safety, Obihiro University of Agriculture and Veterinary Medicine, Obihiro, Hokkaido Japan; 4 Department of Allergy and Clinical Immunology, Graduate School of Medicine, Chiba University, Chiba, Chiba, Japan; 5 Department of Rheumatology, School of Medicine, International University of Health and Welfare, Narita, Chiba Japan

**Keywords:** Fungal host response, Asthma

## Abstract

*Schizophyllum commune* is a ubiquitous basidiomycetous fungus typically found across the world, which has been detected in indoor and outdoor air. Some studies indicated that sensitization to *S. commune* is correlated with asthma severity in patients. Patients with chronic severe or acute fatal asthma have neutrophil-dominant airway inflammation. We hypothesized that *S. commune* can exacerbate asthma. To test this hypothesis, we evaluated the direct immunomodulatory activities of *S. commune* in allergic airway inflammation induced by non-fungal sensitization. Ovalbumin (OVA)-induced asthma model mice were generated using wild-type (WT) and *Il-17a*^−/−^*Il-17f*^−/−^ mice that were intratracheally exposed to *S. commune*, then immune responses in the lungs were assessed after 24 h. Intratracheal administration of *S. commune* in OVA-induced asthma model mice enhanced neutrophilic airway inflammation, increased the mRNA expression of *CXCL1* and *CXCL2* in the lungs, and provoked IL-17A, and IL-17F production in BAL fluid. In addition, neutrophilic airway inflammation was significantly inhibited in *Il-17a*^−/−^*Il-17f*^−/−^ mice compared with those found in WT mice. We demonstrated that *S. commune* induces neutrophilic airway inflammation in OVA-induced asthma model mice, and IL-17A and IL-17F had central roles in this activity. As *S. commune* inhabits the general environment, including indoor and outdoor air, our results suggested that *S. commune* is a causative agent of asthma exacerbation. This study has provided clues regarding the mechanisms behind fungi and asthma exacerbation.

## Introduction

The basidiomycetous fungus *Schizophyllum commune* is typically found in diverse trees and rotting wood across the world^1^. Recent metagenomic analyses of the mycobiome revealed the presence of *Schizophyllum* in indoor and outdoor air^2–4^. In particular, Coombs *et al*. reported that *Schizophyllum* was the third most abundant genus in indoor air samples collected in Cincinnati, Ohio, USA^3^.

*S. commune* causes respiratory allergic diseases, such as allergic bronchopulmonary mycosis^5,6^ and allergic fungal sinusitis^7,8^. Some studies indicated that sensitization to *S. commune* was correlated with asthma severity^9^, and that this sensitization was identified as a risk factor involved in lung function decline in patients with asthma^10^. These studies suggest that pulmonary exposures to S. commune is potentially related to asthma exacerbation.

Asthma is a T helper type 2 (Th2) cell-mediated eosinophilic inflammatory disease, and Th2 cytokines IL-4, IL-5, and IL-13 have been implicated in asthma pathology. However, patients with chronic severe or acute fatal asthma have neutrophil-dominant airway inflammation in addition to Th2-associated airway inflammation^11–13^. Recent studies illustrated that IL-17A and IL-17F, which recruit neutrophils into the airway via the release of CXC chemokines from bronchial epithelial cells, were upregulated in patients with asthma^14^. Elevation of IL-17A and IL-17F levels in the lungs is directly correlated with disease severity^15–19^.

Fungal allergens contain a wide variety of proteins, including proteases, as well as intracellular and extracellular proteins^20,21^. Both protein allergens and fungal cell wall polysaccharides, such as β-glucan, α-mannan, and chitin, have immunomodulatory activities. Schizophyllan (SPG), a cell wall β-glucan derived from *S. commune*, induces the production of proinflammatory cytokines and chemokines regulating neutrophil recruitment^22^, whereas α-mannan induces Th17 cell differentiation through dectin-2^23^. Additionally, elevation of cell wall chitin content enhances the recruitment of lung eosinophils^24–27^. However, the precise mechanism of fungus-induced allergic airway inflammation and its involvement in allergic asthma exacerbation remains unknown.

In the present study, we hypothesized that *S. commune* is potentially associated with asthma exacerbation. To test this hypothesis, we evaluated the direct immunomodulatory activities of *S. commune* in a model of allergic airway inflammation induced by non-fungal sensitization.

## Results

### *S. commune* enhances neutrophilic airway inflammation in OVA-induced asthma model mice

**Figure 1:**
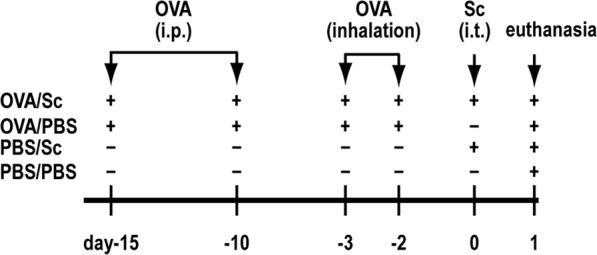
Experimental protocol for OVA-induced asthma model mice and intratracheal administration of *Schizophyllum commune*. Mice were divided into four groups: OVA-sensitized/challenged and intratracheally administered *S. commune* (OVA/Sc), OVA-sensitized/challenged and intratracheally administered PBS (OVA/PBS), non-sensitized and intratracheally administered *S. commune* (PBS/Sc), and non-sensitized and intratracheally administered PBS (PBS/PBS). Mice were intraperitoneally sensitized with OVA on day −15 and day −10, then challenged via exposure to aerosolized OVA on days −3 and −2. Mice in the OVA/Sc and PBS/Sc groups were intratracheally administered a *S. commune* mycelial suspension on day 0. All animals were euthanized 24 h after the intratracheal administration of *S. commune*.

**Figure 2:**
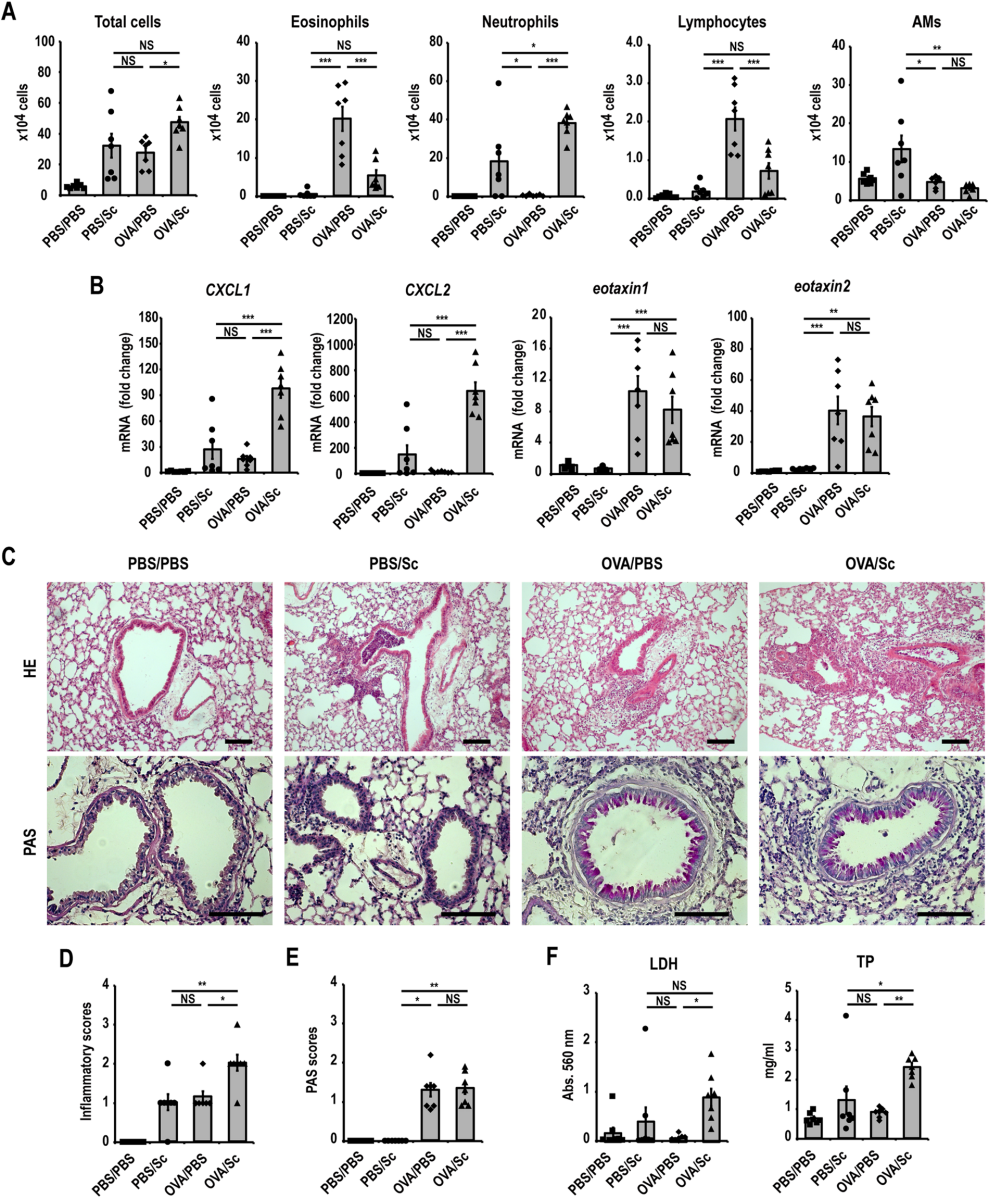
*Schizophyllum commune* enhances neutrophilic airway inflammation in OVA-induced asthma model mice. (**A**) The numbers of total cells, neutrophils, eosinophils, lymphocytes, and alveolar macrophages (AM) in BAL fluid were counted. (**B**) The mRNA expression of *CXCL1*, *CXCL2*, *eotaxin-1* and *eotaxin-2* in the lungs was measured via quantitative real-time PCR. The mRNA levels were normalized to β-actin mRNA levels, then presented as fold differences relative to those in the PBS/PBS group. (**C**) Histological examination of lung tissues was performed via staining with hematoxylin and eosin (HE), and periodic acid-Schiff (PAS) stain. Scale bar, 100 μm. (**D**) Lung inflammation scores. (**E**) PAS scores of airway epithelium graded for goblet cell hyperplasia. (**F**) Lactate dehydrogenase (LDH) activity, and total protein (TP) levels in BAL fluids. All results are expressed as mean ± SEM (n = 7 mice/group). Each symbol represents an individual sample. *P < 0.05, **P < 0.01, ***P < 0.001. NS, not significant.

To investigate the direct immunomodulatory activities of *S. commune* on non-fungus-induced allergic airway inflammation, OVA-induced asthma model mice were intratracheally administered *S. commune* and BAL fluid was collected 24 h after administration (Fig. 1). Intratracheal administration of *S. commune* to OVA-induced asthma model mice (OVA/Sc group) increased the number of neutrophils in BAL fluid (Fig. 2A). On the contrary, the numbers of eosinophils and lymphocytes decreased in the OVA/Sc group (Fig. 2A). We next evaluated the mRNA expression levels of neutrophil (CXCL1 and CXCL2) and eosinophil chemotactic factors (eotaxin-1 and eotaxin-2) in the lungs. The mRNA expression of *CXCL1* and *CXCL2* was increased in the OVA/Sc group (Fig. 2B). On the contrary, *eotaxin-1* and *eotaxin-2* expression was comparable between OVA/Sc and OVA/PBS group (Fig. 2B). Histological examinations revealed that OVA/Sc mice presented with higher lung inflammation scores compared with the PBS/Sc and OVA/PBS groups (Fig. 2C,D). Lung permeability and cellular damage were assessed by evaluating total protein (TP) and lactate dehydrogenase (LDH) activity in BAL fluid, and levels of TP and LDH activity in BAL fluid were elevated in the OVA/Sc group compared with the PBS/Sc and OVA/PBS groups (Fig. 2F). PAS scores reflecting goblet cell hyperplasia in airway epithelium did not vary between the OVA/Sc and OVA/PBS groups (Fig. 2C,E).

### *S. commune* induces Th17-related cytokine production in OVA-induced asthma model mice

**Figure 3:**
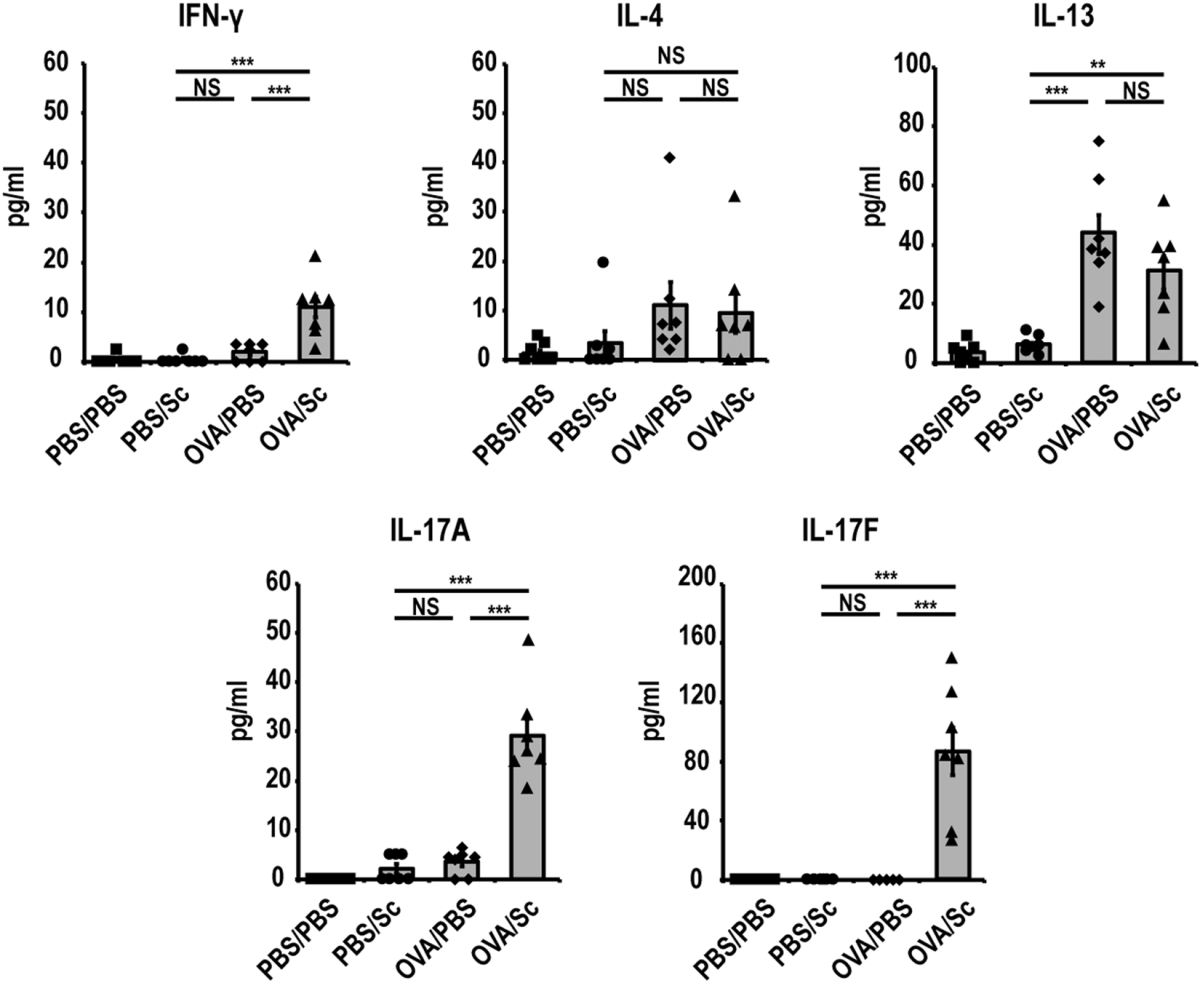
*S. commune* induces Th17-related cytokine production in OVA-induced asthma model mice. IFN-γ, IL-4, IL-13, IL-17A, and IL-17F levels in BAL fluid were measured by ELISA. All results are expressed as mean ± SEM (n = 7 mice/group). Each symbol represents an individual sample. *P < 0.05, **P < 0.01, ***P < 0.001, NS, not significant.

To determine Th1, Th2, and Th17 immune responses in the lungs after *S. commune* administration in OVA-induced asthma model mice, Th1-, Th2- and Th17-related cytokine levels in BAL fluid were measured using ELISA. Intratracheal administration of *S. commune* to the OVA-induced asthma model mice induced the production of the Th17-related cytokines, IL-17A and IL-17F, as well as Th1-related cytokine INF-γ in the lungs (Fig. 3). On the contrary, levels of the Th2-related cytokines IL-4 and IL-13 were comparable between the OVA/Sc and OVA/PBS groups (Fig. 3).

### IL-17A and IL-17F have central roles in neutrophilic airway inflammation induced by *S. commune*

**Figure 4:**
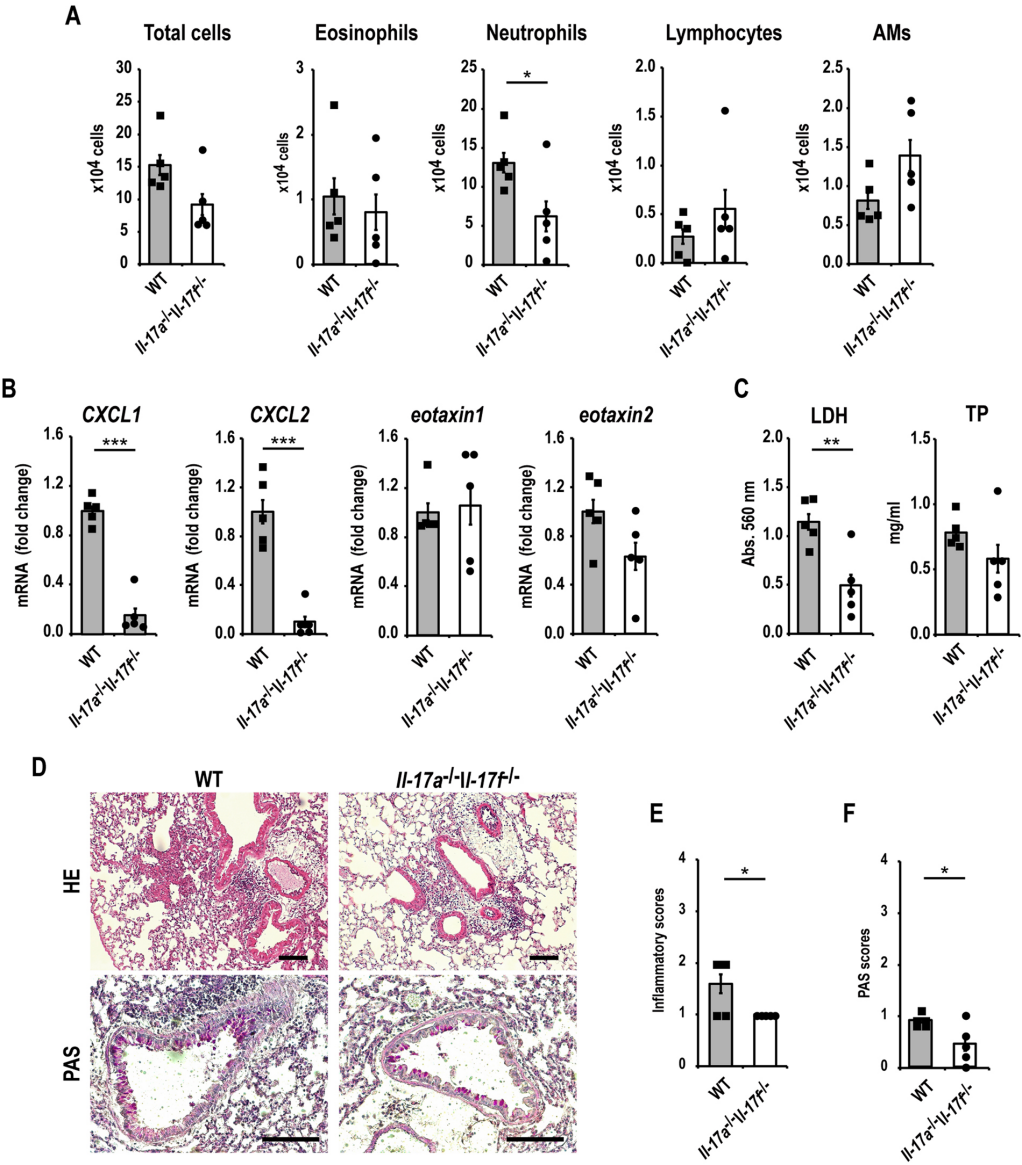
IL-17A/F have central roles in *Schizophyllum commune*-induced neutrophilic airway inflammation in OVA-induced asthma model mice. *Il-17a*^−/−^*Il-17f*^−/−^ and wild-type (WT) mice were sensitized and challenged with OVA, then administered a *S. commune* mycelial suspension as described in Fig. 1. All animals were euthanized 24 h after the administration of *S. commune*. (**A**) The numbers of total cells, neutrophils, eosinophils, lymphocytes, and alveolar macrophages (AM) in BAL fluids were counted. (**B**) The mRNA expression of *CXCL1*, *CXCL2*, *eotaxin-1* and *eotaxin-2* in the lungs was measured via quantitative real-time PCR. The mRNA levels were normalized to β-actin mRNA levels and presented as fold differences relative to those in the WT group. (**C**) Lactate dehydrogenase (LDH) and total protein (TP) levels in BAL fluid. (**D**) Histological examination of lung tissues after staining with hematoxylin and eosin (HE), and periodic acid-Schiff (PAS) stain. Scale bar, 100 μm. (**E**) Lung inflammation scores. (**F**) PAS scores of airway epithelium graded for goblet cell hyperplasia. All results are expressed as mean ± SEM (n = 5 mice/group). Each symbol represents an individual sample. *P < 0.05, **P < 0.01, ***P < 0.001.

IL-17A and IL-17F levels in BAL fluid were clearly increased in the OVA/Sc group (Fig. 3). We next hypothesized that IL-17A and IL-17F were involved in neutrophilic airway inflammation induced by *S. commune* in OVA-induced asthma model mice. To demonstrate this hypothesis, we investigated the roles of IL-17A and IL-17F using OVA-induced asthma model mice, generated by knocking out *IL-17a* and *IL-17f* (*Il-17a*^−/−^*Il-17f*^−/−^), and WT mice. Neutrophilic infiltration in BAL fluid after the intratracheal administration of *S. commune* was reduced in *Il-17a*^−/−^*Il-17f*^−/−^ mice compared with that in WT mice (Fig. 4A). Similarly, the mRNA expression of *CXCL1* and *CXCL2* in the lungs after *S. commune* administration was concomitantly suppressed in these mice (Fig. 4B). Moreover, LDH activity and TP levels in BAL fluid were reduced in *Il-17a*^−/−^*Il-17f*^−/−^ mice (Fig. 4C). Histological examinations revealed that *Il-17a*^−/−^*Il-17f*^−/−^ mice presented with lower lung inflammation and PAS scores than WT mice (Fig. 4D–F).

## Discussion

We found that *S. commune*, a ubiquitous basidiomycetous fungus in the environment, induces neutrophilic airway inflammation in non-fungus-induced asthma model mice and that IL-17A and IL-17F have central roles in the neutrophilic airway inflammation induced by *S. commune*.

Recently, Coombs *et al*. reported that *Schizophyllum* was the third most abundant genus in indoor air samples in Cincinnati, Ohio, USA^3^. Ogawa *et al*. reported that *S. commune* sensitization is correlated with asthma severity^9^ and that it is one of the risk factors involved in lung function decline in patients with asthma^10^.

It is not clear which component of *S. commune* (mycelium, fruiting body, basidiospore) is inhaled. This study used lyophilized mycelium because it is easy to culture and quantify. For intratracheal administration, 100 µg of *S. commune* mycelia (total protein, 80 µg) was used. In previous reports with inhalation mouse models of allergic fungal asthma^28,29^, 20–100 μg of *Aspergillus fumigatus* extract was used to sensitize and challenge the mice. We chose the 100-µg dose of mycelial suspension with reference to these reports.

Meanwhile, IL-17A and IL-17F are highly homologous members of the IL-17 cytokine family, meaning they may bind the same receptor complexes consisting of IL-17RA and IL-17RC^30,31^. IL-17A and IL-17F induce the production of pro-inflammatory cytokines (IL-1, IL-6, TNF-α) and chemokines (CXCL1, CXCL2), thus enhancing neutrophil recruitment^31,32^. Recent studies illustrated the upregulation of IL-17A and IL-17F in asthma and reported that elevation of IL-17A and IL-17F levels in the lungs is directly correlated with disease severity^15–19^. Some studies observed neutrophil-dominant airway inflammation in certain patients with chronic severe or acute fatal asthma^11–13^. In line with our findings, these data suggest that *S. commune* can exacerbate asthma by inducing IL-17A– and IL-17F–mediated neutrophilic airway inflammation.

Some fungal components can trigger IgE-mediated allergies, whereas others are immunomodulators with effects on asthma independent of their potential antigenic activity^33^. Fungal cell wall polysaccharides, such as β-glucan, α-mannan, and chitin, have immunomodulatory activities. β-glucan is recognized by the innate immune receptor dectin-1, while signaling through dectin-1 promotes fungal immunity by stimulating dendritic cells to polarize T cells toward Th17 cells^34,35^. SPG, a cell wall β-glucan derived from *S. commune*, also has immunomodulating potential and antitumor activity^22,36–38^, and it induces the production of IL-6, IL-8, and TNF-α, which regulate neutrophil recruitment^22^. Fungal mannose residues are recognized by dectin-2, macrophage mannose receptor, and DC-SIGN, which are expressed on the surface of macrophages or dendritic cells^23,39,40^. Signaling through dectin-2 promotes the polarization of T cells toward Th17 cells^23^. Chitin induces eosinophilic infiltration in the lungs^41,42^ by inducing the release of epithelial cell-derived cytokines, namely TSLP, IL-25, and IL-33, which activate innate lymphoid type 2 cells^43–45^. In our study, *S. commune* provoked Th17-related cytokine production in OVA-induced asthma model mice. It was presumed that the fungal components inducing Th17 immune responses in OVA-induced asthma model mice might be β-glucan or mannan. However, the precise mechanism of these responses still remains unclear.

In conclusion, we demonstrated that the basidiomycetous fungus *S. commune* induces neutrophilic airway inflammation in OVA-induced asthma model mice, while IL-17A and IL-17F play central roles in neutrophilic airway inflammation induced by *S. commune*. Considering the ubiquitous nature of *S. commune* in the general environment, our results suggested that *S. commune* is a causative agent of asthma exacerbation. These findings provide clues regarding the mechanism behind fungi and asthma exacerbation.

## Materials and Methods

### Preparation of mycelial suspension

This study used a dikaryotic strain of *S. commune* (IFM 47009; Medical Mycology Research Center, Chiba University, Chiba, Japan) isolated from a patient with allergic bronchopulmonary mycosis^46^. After 7 days of culture on potato dextrose agar (PDA) (Becton, Dickinson and Company, New Jersey, USA) at 25 °C, mycelia were inoculated into yeast nitrogen base broth (Becton, Dickinson and Company, New Jersey, USA), supplemented with 1% glucose, and cultured for 5 days at 37 °C with agitation at 200 rpm. The mycelia were collected using Miracloth (Merck Millipore Limited, Massachusetts, USA) and lyophilized after twice washing with phosphate-buffered saline (PBS). Lyophilized mycelia were resuspended in PBS at a final concentration of 2 mg/ml, disrupted by beads beating using a Multibeads Shocker^®^ (Yasui Kikai Co., Osaka, Japan). The inactivation of mycelia was confirmed by culturing the mycelial suspension on PDA plates at 25 °C for 7 days. The total protein concentration of the mycelial suspension was 1.6 mg/ml. The mycelial suspension was subsequently stored at −80 °C until use. A photomicrograph of the mycelial suspension is shown in Fig. S1.

### Animals

Specific pathogen-free female C57BL/6 mice, aged 8 weeks, were purchased from Charles River Laboratories Japan (Yokohama, Japan). Female *Il-17a*^−/−^*Il-17f*^−/−^ mice (C57BL/6 background)^47^, aged 8 weeks, were kindly gifted by Prof. Y. Iwakura (Research Institute for Biomedical Sciences, Tokyo University of Science, Japan). Genotyping of *Il-17a*^−/−^*Il-17f*^−/−^ mice prior to experimentation was performed as described previously^47,48^. All mice were housed under specific pathogen-free conditions with food and water *ad libitum*. All animal experiments were approved by the Committee on Animal Experiments of Chiba University and carried out according to the Chiba University Animal Experimentation Regulations.

### OVA-induced asthma model mice and intratracheal administration of *S. commune*

Mice were divided into four groups as follows: ovalbumin (OVA)-sensitized/challenged and intratracheally administered *S. commune* (OVA/Sc group), OVA-sensitized/challenged and intratracheally administered PBS (OVA/PBS group), non-sensitized and intratracheally administered *S. commune* (PBS/Sc group), and non-sensitized and intratracheally administered PBS (PBS/PBS group). Mice in the OVA/PBS and OVA/Sc groups were intraperitoneally sensitized with 20 µg of OVA (Grade III; Sigma-Aldrich, Missouri, USA) and 2 mg of alum (Thermo Fisher Scientific, Massachusetts, USA) in 0.2 ml of PBS on days −15 and −10, then challenged via exposure to aerosolized 1% OVA (w/v) for 40 min using a nebulizer (PARI Boy N, PARI, Starnberg, Germany) on days −3 and −2. Under anesthesia with ketamine and xylazine, mice in the PBS/Sc and OVA/Sc groups were intratracheally administered 100 µg of *S. commune* mycelia suspended in 50 µl of PBS on day 0. All animals were euthanized 24 h after the intratracheal administration of *S. commune* (Fig. 1).

### Total and differential leukocyte counts in BAL fluid

Airway contents were recovered via the instillation and retrieval of 2 ml of sterile PBS. The lavage fluid was centrifuged, and the cell pellet was resuspended in PBS. Total cell numbers were quantified with a hemocytometer under a light microscope. Cells were centrifuged onto glass slides using Cytospin™ (Thermo Fisher Scientific) and stained with Diff-Quick (Wako Chemicals, Osaka, Japan) for differential counts of leukocytes. A total of 300 cells were counted on each slide.

### TP and LDH levels in BAL fluid

The TP level in BAL fluid was measured using a BCA Protein Assay Reagent Kit (Thermo Fisher Scientific). LDH activity in BAL fluid was measured using the LDH-Cytotoxic Test (Wako Chemicals).

### Cytokine levels in BAL fluid

IFN-γ, IL-17A, IL-17F, IL-13, and IL-4 levels in BAL fluid were measured using enzyme-linked immunoassay (ELISA) kits (R&D Systems, Minnesota, USA), according to the manufacturer’s instructions.

### Lung histopathology

Mouse lungs were fixed in 4% formaldehyde, routinely embedded in paraffin, and sectioned at a thickness of 4 µm. Sections were stained separately with hematoxylin and eosin and periodic acid-Schiff (PAS) stain. Inflammation was graded using a 0–4 grade scoring system: 0, no inflammation; 1, mild inflammation; 2, moderate inflammation; 3, severe inflammation; and 4, extreme inflammation, as described previously^49^. Hyperplasia of goblet cells in the epithelial samples was assessed via PAS staining using a 0–4 grade scoring system. Ten bronchi in the lungs were examined, and average scores were calculated. Each bronchus was graded as follows: 0, no PAS-positive cells; 1, 1%–25% PAS-positive cells; 2, 26%–50% PAS-positive cells; 3, 51%–75% PAS-positive cells; and 4, 76%–100% PAS-positive cells, as described previously^50^.

### mRNA extraction and quantitative real-time PCR

Mouse lungs were fixed in RNAlater® (Thermo Fisher Scientific) and stored at −20 °C. Fixed lungs were homogenized using a Multibeads Shocker^®^ and total RNA was isolated using RNAiso plus (Takara Bio, Shiga, Japan) and Zymo-Spin II (Zymo Research, California, USA), according to the manufacturers’ instructions. The purity of total RNA was checked using a NanoDrop 1000 (Thermo Fisher Scientific) and agarose gel electrophoresis. cDNA was generated via reverse transcription using PrimeScript RT Master Mix (Takara Bio). Quantitative real-time PCR was performed using SYBR^®^ Premix Ex Taq™ II (Takara Bio) and the following primer pairs: *CXCL1* (Fwd, 5′-TGGCTGGGATTCACCTCAAG-3′, Rev, 5′-CAGACAGGTGCCATCAGAGC-3′); *CXCL2* (Fwd, 5′-AGACAGAAGTCATAGCCACTCTCAAG-3′, Rev, 5′-CCTCCTTTCCAGGTCAGTTAGC-3′); *eotaxin-1* (Fwd, 5′-TCCACAGCGCTTCTATTCCT-3′, Rev, 5′-CTATGGCTTTCAGGGTGCAT-3′); *eotaxin-2* (Fwd, 5′- GCTGCACGTCCTTTATTTCC-3′, Rev, 5′- TCTTATGGCCCTTCTTGGTG-3′); and *β-actin* (Fwd, 5′-GCTGTATTCCCCTCCATCGTG-3′, Rev, 5′-CACGGTTGGCCTTAGGGTTCAG-3′). Real-time PCR amplification was performed using an Applied Biosystems 7300 Real-Time PCR System (Thermo Fisher Scientific) under the following conditions: one cycle at 95 °C for 30 s, followed by 40 cycles at 95 °C for 5 s and 60 °C for 31 s. mRNA levels were normalized to those of β-actin and presented as fold changes, relative to the PBS/PBS or WT group.

### Statistical analysis

Statistical analysis was performed using GraphPad InStat 3 and JMP^®^7. Unpaired, two-tailed Student’s *t*-tests and one-way ANOVA with *post-hoc* Tukey–Kramer tests were used to assess statistical significance. P < 0.05 was considered to indicate a significant difference.

## Supplementary information

Supplementary Information

## Data Availability

The datasets during and/or analysed during the current study available from the corresponding author on reasonable request.
