# Supplementary material for: Schizophyllum commune induces IL-17-mediated neutrophilic airway inflammation in OVA-induced asthma model mice
Source: Sci Rep. 2019 Dec 18;9:19321. doi: 10.1038/s41598-019-55836-x (PMC6920419; doi:10.1038/s41598-019-55836-x)
Supplement: Supplementary file 1 — Supplementary Information [file 41598_2019_55836_MOESM1_ESM.pdf]

Schizophyllum commune induces IL-17-mediated neutrophilic airway inflammation in OVA-induced asthma model mice

Jun Hanashiro[1*], Yasunori Muraosa[1*#], Takahito Toyotome[2, 3, 1], Koichi Hirose[4,5], Akira Watanabe[1], Katsuhiko Kamei[1]

[*]Both authors contributed equally to this work.

[1] Division of Clinical Research, Medical Mycology Research Center, Chiba University, Chiba, Chiba, Japan.

[2] Department of Veterinary Medicine, Obihiro University of Agriculture and Veterinary Medicine, Obihiro, Hokkaido, Japan

[3] Diagnostic Center for Animal Health and Food Safety, Obihiro University of Agriculture and Veterinary Medicine, Obihiro, Hokkaido, Japan.

[4] Department of Allergy and Clinical Immunology, Graduate School of Medicine, Chiba University, Chiba, Chiba, Japan.

[5] Department of Rheumatology, School of Medicine, International University of Health and Welfare, Narita, Chiba, Japan.

[#] Correspondence: E-mail: y.muraosa@faculty.chiba-u.jp

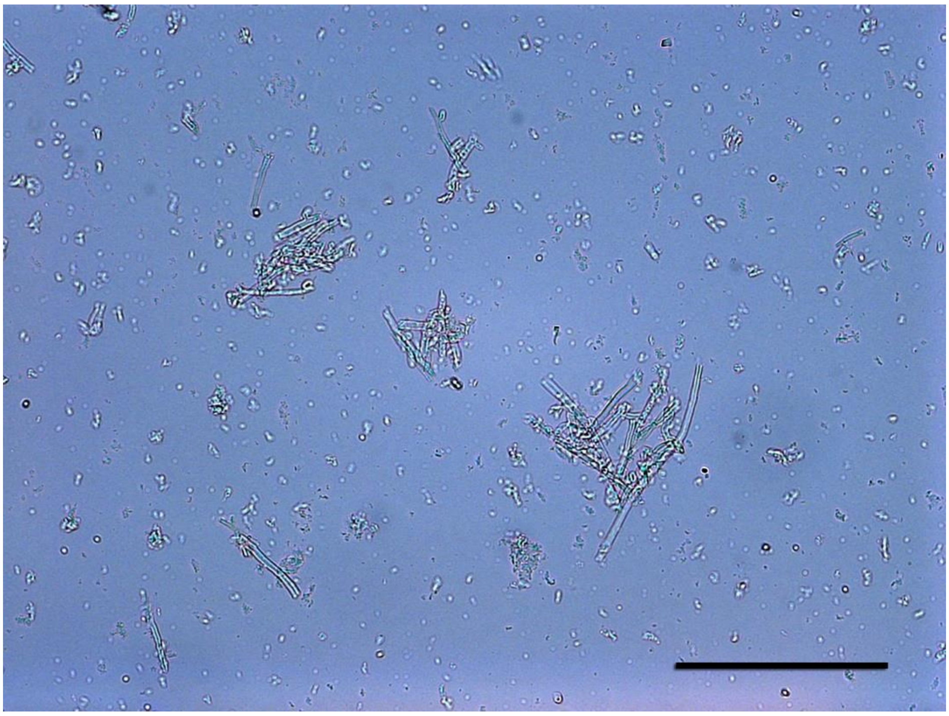

Supplementary Figure S1. Photomicrograph of *Schizophyllum commune* mycelial suspension. Bar, 100 μm.
